# Circulating microRNAs in follicular fluid, powerful tools to explore *in vitro* fertilization process

**DOI:** 10.1038/srep24976

**Published:** 2016-04-22

**Authors:** E. Scalici, S. Traver, T. Mullet, N. Molinari, A. Ferrières, C. Brunet, S. Belloc, S. Hamamah

**Affiliations:** 1 CHU Montpellier, Institute of Regenerative Medicine and Biotherapy, Saint-Eloi Hospital, INSERM U1203, Montpellier, France; 2 Montpellier 1 University, UFR of Medicine, Montpellier, France; 3 ART-PGD Department, Arnaud de Villeneuve Hospital, CHU Montpellier, Montpellier, France; 4 UMR 1049, DIM, CHRU, Montpellier, France; 5 Eylau/Unilabs Laboratory, Reproductive Biology Unit, 55 Rue Saint Didier, 75016 Paris, France

## Abstract

Circulating or “extracellular” microRNAs (miRNAs) detected in biological fluids, could be used as potential diagnostic and prognostic biomarkers of several disease, such as cancer, gynecological and pregnancy disorders. However, their contributions in female infertility and *in vitro* fertilization (IVF) remain unknown. This study investigated the expression profiles of five circulating miRNAs (let-7b, miR-29a, miR-30a, miR-140 and miR-320a) in human follicular fluid from 91 women with normal ovarian reserve and 30 with polycystic ovary syndrome (PCOS) and their ability to predict IVF outcomes. The combination of FF miR-30a, miR-140 and let-7b expression levels discriminated between PCOS and normal ovarian reserve with a specificity of 83.8% and a sensitivity of 70% (area under the ROC curve, AUC = 0.83 [0.73–0.92]; p < 0.0001). FF samples related to low number of mature oocytes (≤2) contained significant less miR-320a levels than those related to a number of mature oocytes >2 (p = 0.04). Moreover, FF let-7b predicted the development of expanded blastocysts with 70% sensitivity and 64.3% specificity (AUC = 0.67 [0.54–0.79]; p = 0.02) and FF miR-29a potential to predict clinical pregnancy outcome reached 0.68 [0.55–0.79] with a sensitivity of 83.3% and a specificity of 53.5% (p = 0.01). Therefore, these miRNAs could provide new helpful biomarkers to facilitate personalized medical care during IVF.

MicroRNAs (miRNAs) are single stranded, small non-coding RNA molecules of 19–25 nucleotides in length that contribute to post-transcriptional regulation by binding specifically to messenger RNA targets, leading to their destabilization or translation repression^1^. In reproduction, they are involved in the regulation of several biological processes, such as follicular cell proliferation and apoptosis^2^,^3^, steroidogenesis^4^,^5^ and oocyte maturation^3^,^6^. Among the miRNAs expressed in ovarian follicles, some are also found in the bloodstream and other biological fluids, such as follicular fluid (FF)^5^,^7^,^8^. These circulating/extracellular miRNAs, can be easily quantified in biological fluids^9^ and might represent promising biomarkers in some medical fields such as oncology^10^,^11^, obstetrics^12^,^13^ and gynecology^13^,^14^, due to their possible applications in the diagnosis and prognosis of many diseases^15^.

Currently, the ovarian reserve status is routinely determined based on the serum concentration of anti-Müllerian hormone (AMH) and the antral follicle count (AFC); however, this approach is confronted with a lack of standardization among laboratories and practitioners, respectively^16^,^17^,^18^. Few studies have provided evidence that miRNA expression is altered in serum or in FF samples from women with polycystic ovary syndrome (PCOS)^5^,^19^,^20^,^21^,^22^,^23^. Therefore, analysis of the differential expression of specific miRNAs in FF samples from women with PCOS could represent a promising approach to identify novel specific biomarkers for the assessment of ovarian reserve status. Moreover, the ovarian response to controlled ovarian stimulation (COS) protocols is affected by the ovarian reserve status and influences *in vitro* fertilization (IVF) outcomes^24^. Although, it was reported that some miRNAs are implicated in the regulation of steroidogenesis in ovarian follicles^4^,^5^,^25^,^26^, the impact of gonadotropin treatments on miRNA expression in FF remains unknown.

FF also provides a micro-environment that influences the oocyte developmental competence and embryo development. Consequently, variations in its content have been considered as predictive factors of oocyte and embryo quality^27^,^28^,^29^. Moreover, it was demonstrated that miRNAs play a role in oocyte and embryo development^30^,^31^. Although one recent study suggested the association between miR-320a expression in FF and embryo quality^32^, the use of the expression profile of circulating miRNAs in the oocyte micro-environment as a tool to predict blastocyst development and clinical pregnancy outcomes during IVF has never been investigated.

In this study, the expression profiles of five circulating miRNAs (let-7b, miR-29a, miR-30a, miR-140 and miR-320a) were compared in FF samples from women with normal ovarian reserve and with PCOS, undergoing IVF/Intra-cytoplasmic sperm injection (ICSI) procedure, with the aim of identifying new efficient biomarkers related to PCOS. We also assessed whether their differential expression was influenced by the COS protocol and ovarian response. Finally, we tested the performance of these FF miRNAs to predict blastocyst formation and clinical pregnancy outcome, in women with normal ovarian reserve. Our results indicate that some of these miRNAs are differentially expressed in FF samples from women affected by PCOS, suggesting that their specific profile could be used to discriminate these patients. Moreover, we demonstrate, for the first time, that the miRNA content in FF could be related to gonadotropin treatment and ovarian response. In addition, some circulating miRNAs could constitute non-invasive powerful tools for the prediction of blastulation and clinical pregnancy outcome towards the development of a personalized IVF strategy.

## Results

### MiRNA differential expression in FF samples from women with PCOS compared to women with normal ovarian reserve

**Figure 1:**
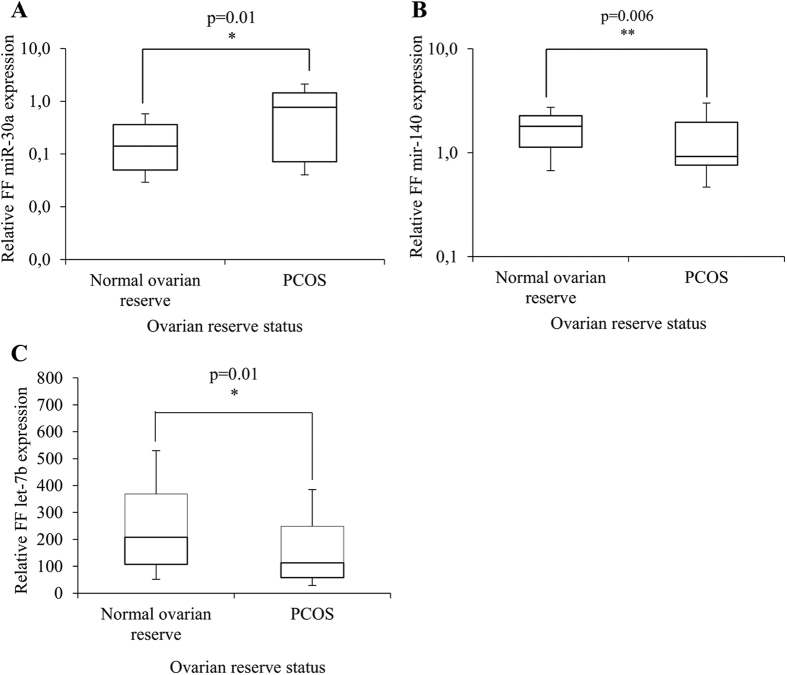
Comparison of the relative miRNA expression in follicular fluid (FF) pools from women with normal ovarian reserve (n = 91) and with polycystic ovary syndrome (PCOS, n = 30). (**A**) FF miR-30a; (**B**) FF miR-140; (**C**) FF let-7b. P-values: Mann-Whitney test.

**Figure 2:**
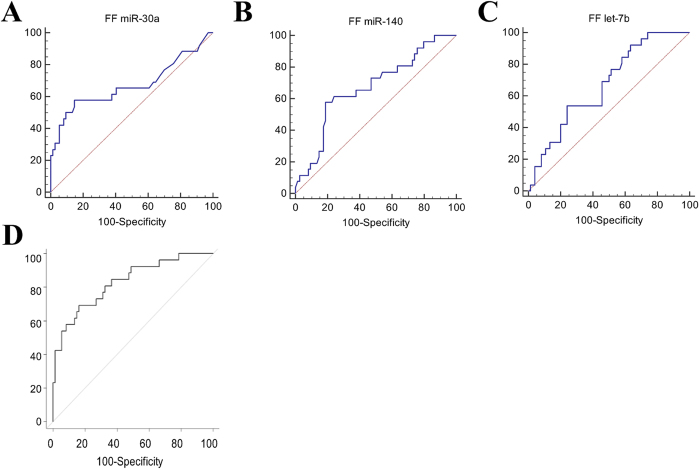
ROC curve analysis to calculate the discriminative power of the FF expression of (**A**) miR-30a; (**B**) miR-140; and (**C**) let-7b individually and (**D**) in combination to predict PCOS.

Comparison of the expression profiles showed that miR-30a was significantly up-regulated (p = 0.006), while miR-140 and let-7b were significantly down-regulated (p = 0.01 for both) in FF pools from patients with PCOS (n = 30) compared to women with normal ovarian reserve (n = 91) (Fig. 1A–C). Moreover, after adjustment for body mass index (BMI), these three miRNAs were significantly and independently associated with PCOS in multivariate analysis (adjusted odds ratio, AOR: 5.0 [1.86; 13.68], p = 0.001; 0.52 [0.29; 0.94], p = 0.03; 1.0 [0.99; 1.0], p = 0.02, respectively) (Table 1). Then, the sensitivity and specificity of the relationship between FF miR-30a, miR-140 and let-7b differential expression and PCOS were determined using the Receiver Operating Characteristic (ROC) curve analysis and by calculating the area under the ROC curve (AUC). The AUC values for the individual performance of FF miR-30a, FF miR-140 and FF let-7b expression profiles in PCOS discrimination were 0.67 (0.57–0.76), 0.67 (0.57–0.76) and 0.67 (0.57–0.76) (p = 0.02, p = 0.007, p = 0.003), respectively (Fig. 2A–C, Table 2). By combining the three miRNAs in multivariate analysis, the AUC value increased to 0.83 (0.73–0.92) (p < 0.0001) (Fig. 2D, Table 2). Moreover, the sensitivity and the specificity of FF miR-30a, FF miR-140 and FF let-7b were 57.7%, 57.7% and 53.9% and 85.1%, 81.1% and 75.7%, respectively (Table 2). The combination of these three miRNAs increased the sensitivity of the prediction to 70% with a specificity of 83.8%. These results indicate that the combination of miR-30a, miR-140 and let-7b, which are differentially expressed in FF samples from patients with PCOS compared to women with normal ovarian reserve, gives the largest AUC value with high sensitivity and specificity, and suggest that these three miRNAs represent new potential PCOS biomarkers.

### Differential expression of FF miRNAs according to the gonadotropin treatment and ovarian response

The variations of miRNA expression in FF samples were investigated relative to the gonadotropin treatment and ovarian response in women with normal ovarian reserve (n = 91).

**Figure 3:**
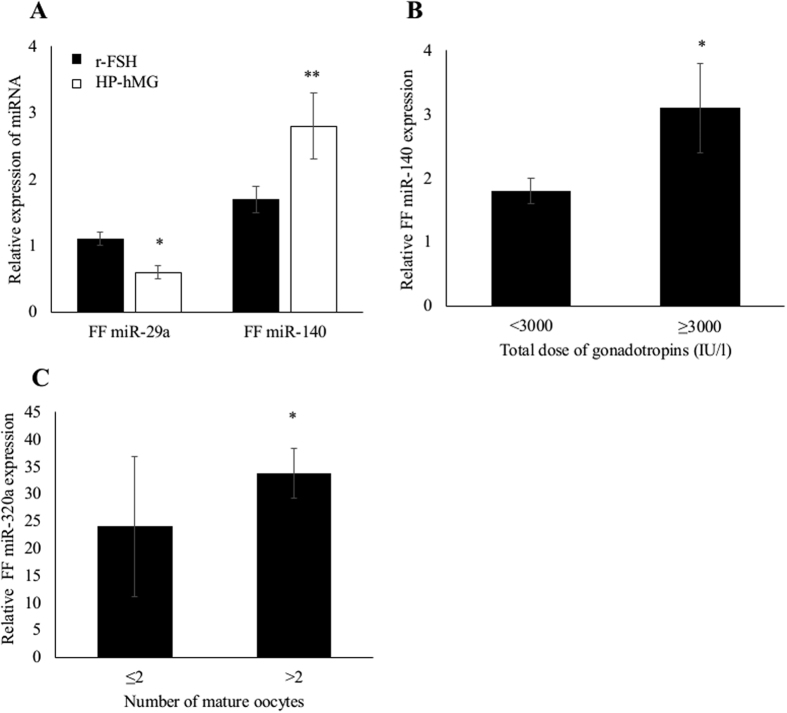
(**A**) Comparison of FF miR-29a and miR-140 expression level relative to the type of treatment (highly purified human menopausal gonadotropin, HP-hMG, versus recombinant follicle-stimulating hormone, r-FSH) (*p = 0.03; **p = 0.02). (**B**) Differential FF miR-140 expression according to the total dose of gonadotropins (<3000 versus ≥3000 IU/l) (*p = 0.03). (**C**) Comparison of FF miR-320a expression level relative to the number of retrieved mature oocytes (≤2 versus >2 mature oocytes) (*p = 0.04). These analyses included only the group of women with normal ovarian reserve (n = 91). P-values: Mann-Whitney test.

The expression of the five miRNAs was comparable in FF pools from women who received agonist or antagonist protocols. Conversely, FF expression of miR-29a and miR-140 varied significantly according to the gonadotropin treatment. Specifically, miR-29a expression was significantly decreased and miR-140 expression significantly increased in FF pools from women treated with highly purified human menopausal gonadotropin (HP-hMG) compared with patients who were stimulated with recombinant follicle-stimulating hormone (r-FSH) (p = 0.03; p = 0.02, respectively) (Fig. 3A). Moreover, whatever the type of gonadotropin, miR-140 was significantly up-regulated in FF pools from women who received higher total doses of gonadotropins (≥3000 IU/l) compared to those treated with lower doses (<3000 IU/l) (p = 0.03) (Fig. 3B). Likewise, Spearman’s correlation analysis showed that FF miR-140 level was significantly and positively associated with the total dose of gonadotropins (r = 0.21; p = 0.02) (data not shown).

At oocyte retrieval day, FF pools from women with a low number of mature oocytes (MII) (≤2) contained significant lower FF miR-320 levels than those related to a number of mature oocytes higher than 2 (p = 0.04) (Fig. 3C). By using Spearman’s correlation analysis, miR-320a level in FF pools was significantly and positively correlated with the number of mature oocytes (r = 0.24; p = 0.02) (data not shown).

### FF let-7b expression and blastocyst development

**Figure 4:**
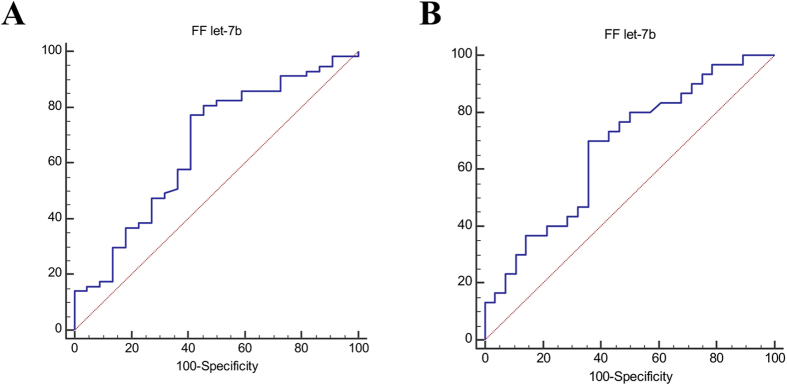
(**A**) ROC analysis to evaluate FF let-7b expression predictive value for blastocyst formation. (**B**) ROC analysis to evaluate FF let-7b expression predictive value for expanded blastocyst development. These analyses included only the group of women with normal ovarian reserve (n = 91).

By considering only the group of women with normal ovarian reserve (n = 91), we found a significant and negative correlation between FF let-7b expression level and blastulation rate (r = −0.33, p = 0.003) Indeed, low FF let-7b expression was significantly associated with the probability to obtain a blastocyst [crude odds ratio, COR = 1.0 [0.99; 1.0], p = 0.04] (data not shown). The AUC value of FF let-7b potential to predict blastocyst development, was 0.66 (0.55–0.76) with 77.2% sensitivity and 59.1% specificity (p = 0.02; at cut-off value ≤ 273.2) (Fig. 4A). Likewise, FF let-7b levels were also correlated significantly and negatively with the expanded blastocyst rate in women with normal ovarian reserve (r = −0.28, p = 0.009). The probability to obtain an expanded blastocyst was significantly associated with intra-follicular expression of let-7b [COR = 1.0 [0.99; 1.0], p = 0.02] (data not shown). In addition, the AUC value that defined the performance of FF let-7b in predicting the formation of expanded blastocysts was 0.67 (0.54–0.79), with 70% sensitivity and 64.3% specificity (p = 0.02; at cut-off value ≤ 247.9) (Fig. 4B).

### FF miR-29a predictive value for clinical pregnancy outcome

**Figure 5:**
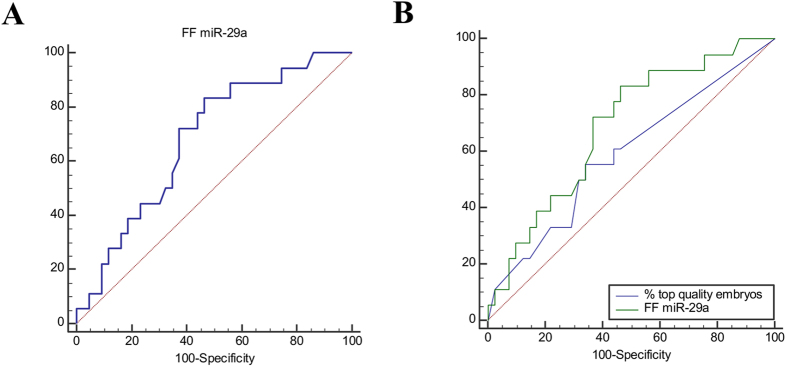
(**A**) ROC analysis to evaluate FF miR-29a expression predictive value for clinical pregnancy outcome. (**B**) Comparison of the ROC curves showing the predictive value of FF miR-29a expression and top quality embryo proportion for clinical pregnancy outcome. These analyses included only the group of women with normal ovarian reserve (n = 91).

In the group with normal ovarian reserve (n = 91), FF miR-29a expression predicted significantly the clinical pregnancy outcome [COR = 2.08 [1.0; 4.3], p = 0.049] (data not shown). Moreover, the ROC curve analysis indicated that the performance of FF miR-29a for clinical pregnancy prediction reached 0.68 (0.55–0.79) with a sensitivity of 83.3%, but a low specificity (53.5%) (p = 0.01; cut-off value > 0.32) (Fig. 5A). In addition, comparison of the discrimination power of FF miR-29a expression and of the top quality embryo percentage for clinical pregnancy prediction showed that the AUC value related to FF miR-29a expression was higher than that for the top quality embryo percentage (AUC = 0.59 [0.46–0.72]; p = 0.27) (Fig. 5B).

## Discussion

**Figure 6:**
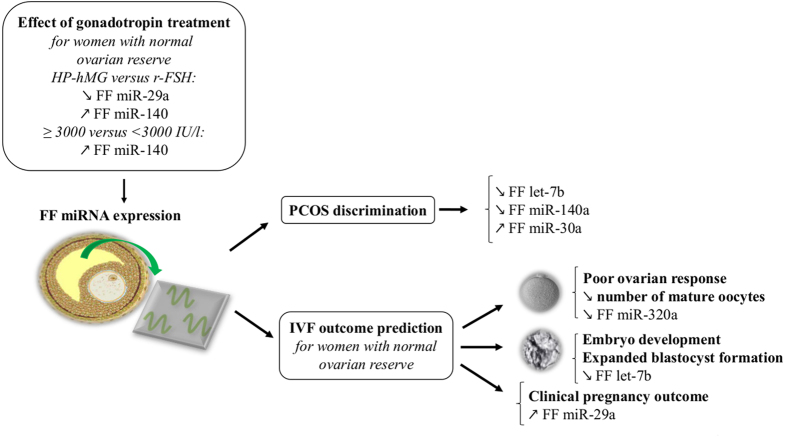
Schematic model showing that miRNA expression profiling in FF samples provides powerful tools to efficiently discriminate women with polycystic ovary syndrome (PCOS) and to predict IVF outcomes. The expression of some FF miRNAs varies according to the gonadotropin treatment. HP-hMG, highly purified human menopausal gonadotropin; r-FSH, recombinant follicle-stimulating hormone.

This study investigated the expression profiles of five circulating miRNAs (let-7b, miR-29a, miR-30a, miR-140 and miR-320a) in FF pools from patients undergoing IVF/ICSI procedure. These circulating microRNAs were differentially expressed according to the women’s ovarian reserve status, gonadotropin treatments and/or IVF outcomes (Fig. 6).

We demonstrate, for the first time, that the expression of let-7b and miR-140 is significantly decreased whereas miR-30a is up-regulated in FF samples from patients with PCOS. Moreover, the combination of these three miRNAs is significantly associated with PCOS, with high specificity and sensitivity. Therefore, they could constitute new specific biomarkers to easily and efficiently identify women with PCOS. Previous studies reported that let-7b is expressed in granulosa and cumulus cells in mammalian and also human ovaries^3^,^33^,^34^,^35^,^36^,^37^. PCOS is characterized by follicular development abnormalities, suggesting that the normal “dialogue” between oocyte and granulosa cells in early growing follicles might be altered^38^. Accordingly, the significant decrease of FF let-7b expression observed in patients with PCOS might reflect this abnormal folliculogenesis. Indeed, it has been reported that let-7b could play a specific role in ovarian follicular development^33^,^35^,^37^. Specifically, let-7b regulates the TGF-β signaling pathway in goat ovary by targeting the activin receptor І and Smad2/3 genes^35^. TGF-β dysregulation contributes to reproductive abnormalities in PCOS, such as follicle development perturbation^39^. Consequently, let-7b down-regulation in ovarian follicles could lead to TGF-β signaling pathway deregulation and ultimately contribute to PCOS development. Abnormal estrogen receptor (ER) expression could also contribute to poor follicular development and ovulatory failure in PCOS^40^. MiR-140 plays a role as tumor suppressor and is down-regulated in breast cancer via ERα signaling^41^. These findings suggest that the modifications of ERα expression observed in PCOS might influence negatively miR-140 expression in ovarian follicles. Finally, it has been demonstrated that miR-30a overexpression in cultured human granulosa cells promotes BCL2A1, IER3 and cyclin D2 expression by repressing FOXL-2^42^. *FOXL-2* encodes a forkhead transcription factor that is essential for ovarian development^43^. *FOXL-2* conditional knockout in mouse results in sex-reversed follicles with characteristics of cystic follicles, including elevated androgen production by theca cells and morphological transformation of granulosa cells, like in PCOS^44^,^45^. Moreover, androgen-induced hirsutism, described in patients with PCOS, is also observed in women carrying *FOXL-2* mutations^46^. Based on these observations, we hypothesize that miR-30 overexpression in FF pools from women with PCOS might lead to FOXL-2 inhibition/down-regulation in ovarian follicles, thus promoting PCOS symptom development. Differently from two previous study^5^,^47^, FF miR-320a expression was not affected in our group of women with PCOS. However, miR-320a expression level was significantly lower in FF pools from women with less than two mature oocytes (≤2) compared with women with more than two mature oocytes. In the mouse, miR-320a knockdown in oocytes decreased significantly the proportion of mature oocytes that developed into embryos^32^. Taken together, these data suggest that miR-320a is indicative of mature oocyte quantity and quality and that its intra-follicular expression could be modulated by the ovarian response quality of patients undergoing IVF.

MiR-29a was significantly down-regulated and miR-140 overexpressed in FF pools from women who were stimulated with HP-hMG compared with those treated with r-FSH. This is in agreement with a previous study showing that miR-29a is significantly down-regulated by FSH treatment in cultured rat granulosa cells, thus influencing progesterone production^48^. Our data suggest that gonadotropin treatments could affect intra-follicular miRNA expression and ultimately IVF efficacy. We also found that total high dose of gonadotropins was associated with miR-140 up-regulation. This probably reflects a potential dose-effect relationship of gonadotropins on FF miR-140 expression profile.

The importance of miRNAs in early embryo development has been demonstrated in many mammalian species^49^. Although Feng *et al*.^32^, did not observe significant differential expression of let-7b in FF samples according to embryo quality, we found that FF let-7b level was significantly related to the embryo developmental potential. Indeed, let-7b levels in FF predicted significantly blastocyst formation and expansion. It was previously shown that let-7 can regulate developmental timing in *Caenorhabditis elegans*^50^. Therefore, evaluation of FF let-7b expression could be useful to define the best strategy of embryo culture during IVF.

In addition, FF miR-29a levels predicted significantly the clinical pregnancy outcome with higher sensitivity (83.3%) compared to the top quality embryos proportion in our cohort. MiR-29a is highly expressed in rat uterus during embryo implantation and its expression is regulated by blastocyst activation and uterine decidualization^51^. Interestingly, miR-29a expression might influence pregnancy outcome by acting both on the follicular and endometrial side, supporting the hypothesis that favorable follicular and endometrial environments are necessary for conception.

In conclusion, miRNA expression profiling in human FF samples might provide biomarkers to efficiently discriminate women with PCOS and to predict blastocyst development and clinical pregnancy outcomes. However, their specific role in the oocyte micro-environment, their regulation by gonadotropins and their involvement in female infertility should be further investigated. These new potential biomarkers could be used in the daily practice to improve personalized IVF strategies and to identify new therapeutic targets in female infertility management.

## Methods

### Patients

This prospective study included 121 women who underwent conventional IVF (n = 28) or ICSI (n = 93) at the ART-PGD Department of the University Hospital of Montpellier, France. Their mean age was 33.7 ± 4.5 years (mean ± SD; range: 19 to 43 years) and the BMI was 24.1 ± 4.5 kg/m^2^ (mean ± SD; range: 17 and 37.5 kg/m^2^) (Supplementary Table S1). The infertility length was 3.6 ± 1.6 years (mean ± SD) and infertility was primary in 71 couples and secondary in the other 50. Male, female and mixed factors were detected in 32.2%, 38.0% and 23.1% of cases, respectively, while infertility was unexplained in 6.7% of couples. This was the first IVF or ICSI attempt for 38.0% of them, while 62.0% had already undergone at least one cycle (mean number of cycles ± SD: 2.1 ± 1.2). Among the 121 women, 91 had a normal ovarian reserve, based on the serum AMH level and AFC, evaluated at day 3 of the menstrual cycle. PCOS was diagnosed in the remaining 30 women, according to the Rotterdam criteria^52^ (i.e., oligo-and/or anovulation, hyperandrogenism signs and polycystic ovary morphology on ultrasound examination). Basal follicle-stimulating hormone (FSH), luteinizing hormone (LH) and 17βestradiol (E2) serum levels were also measured in each patient at day 3 of the menstrual cycle. The clinical characteristics of all women and in the two groups (normal ovarian reserve and PCOS) are detailed in Supplementary Table S1.

Patients were informed about FF sample collection/analysis and they gave their written informed consent on oocyte retrieval day. This study was approved by the Ethical Committee of the Institute for Regenerative Medicine and Biotherapy and the methods were carried out in accordance with the approved guidelines.

### IVF procedure

A gonadotropin-releasing hormone (GnRH) agonist (Decapeptyl, IpsenPharma) was administered daily to 58 women and an antagonist protocol was used in 59. The remaining four patients received a mild treatment and were thus excluded from the analysis concerning the treatment effect on miRNA expression. These two protocols included COS with two types of gonadotropins: r-FSH (Puregon, MSD, Courbevoie, France or GonalF, Merck Serrono, Lyon, France), or HP-hMG (Menopur, Ferring, Gentilly, France). COS duration was 10.2 ± 1.5 days and the total gonadotropin dose was 2086.6 ± 809.4 IU/l (mean ± SD) (Supplemental Table S1). The ovarian response to stimulation was monitored by measuring the serum E2 concentration and by ultrasound assessment of follicular and endometrial growth. Ovulation was triggered with an injection of 250 μg human chorionic gonadotropin (hCG) (Ovitrelle, Merck Serono, Lyon, France) when at least three follicles reached the diameter of 17 mm or more on ultrasound examination. At ovulation triggering day, the hormonal ovarian response was also evaluated by quantifying serum E2, LH and progesterone levels (Supplementary Table S1).

Oocytes were retrieved by transvaginal ultrasound-guided aspiration 36 h after hCG injection. For each patient, all follicles were aspirated without flushing, cumulus-oocyte complexes were isolated for conventional IVF or ICSI and all FF samples were collected.

Before intracytoplasmic sperm microinjection, the oocyte maturity rate (76%) was assessed after denudation. On average, 9.1 ± 4.5 oocytes (mean ± SD) and 7.1 ± 3.9 mature oocytes (mean ± SD) were collected per patient (Supplemental Table S2). Oocytes were cultured individually in 30 μl micro-droplets of culture medium (Vitrolife) under oil at 37 °C in 5% O_2_, 6% CO_2_ and 89% N_2_, in humid atmosphere. The presence of two pronuclei and two polar bodies, 18–20 h after microinjection or insemination confirmed that the cultured oocytes were normally fertilized (overall fertilization rate = 64%). For each patient, 4.7 ± 3.5 embryos were obtained from the fertilized oocytes at day 2. Among these embryos, 1.7 ± 1.9 were cleaved early at 25 or 27 h after microinjection or insemination, respectively. On day 3, embryo quality was assessed based on morphological criteria (blastomere number, blastomere regularity and fragmentation rate). On average, 1.4 ± 1.8 embryos/patient (mean ± SD) were considered as top quality because they contained 6–8 regular blastomeres and less than 20% fragments. One or two top quality embryos were transferred in utero at day 3, whereas the others were further cultured up to day 5. Blastocysts were classified according to the scoring system developed by Gardner^53^. At day 5, only expanded blastocysts (classified as grade 4 or 5) with inner cell mass and trophectoderm scored as A or B were vitrified using a closed system, following the procedure recommended by Irvine Scientific. Four weeks after embryo transfer, clinical pregnancy was confirmed by the observation of at least one gestational sac and of embryonic heart activity on ultrasound examination. The IVF/ICSI outcomes of all women and in the two groups (normal ovarian reserve and PCOS) are reported in Supplementary Table S2.

### FF sample preparation

At oocyte retrieval day, all FF samples of a patient were collected and pooled (n = 121 pools). A volume of 15 ml from each pool was centrifuged at 3000 g for 15 min. Then, supernatants were removed, filtered through 0.45 μm filters to eliminate cell debris and stored at −80 °C.

### MiRNA extraction

The QIAamp^®^ Circulating Nucleic Acid kit (ref. 55114; Qiagen) was used for isolation and purification of circulating miRNAs from 3 ml of each FF pool according to the manufacturer’s protocol. Briefly, 3 ml of FF pool, 400 μl of Qiagen Proteinase K and 4.2 ml of buffer ACL were mixed by pulse vortexing and incubated at 60 °C for 30 min. After incubation, 9 ml of buffer ACB was added to the lysate and mixed by pulse vortexing. The mixture was then transferred in a QIAamp Mini column by vacuum pressure to adsorb the miRNAs onto a small silica membrane. Next, each membrane was washed in three steps to remove residual contaminants. Highly pure circulating microRNAs were eluted within 40 μl of buffer AVE.

### FF miRNA expression analysis by RT-qPCR

Complementary DNA (cDNA) was generated using the TaqMan MicroRNA reverse transcription kit and miRNA-specific stem-loop primers for let-7b, miR-29a, miR-30a, miR-140 and miR-320a (ref. 4427975, Life Technologies). The 15 μl reaction mix contained 5 μl of sample, 0.15 μl of 100 mM dNTP, 1.5 μl of 10 × RT Buffer, 1 μl of MultiScribe RT enzyme (50U/μl), 0.19 μl of RNase inhibitor (20U/μl), 4.16 μl of nuclease-free water and 3 μl of Taqman RT primer. Reverse transcription was carried out at 16 °C for 30 min and then at 42 °C for 30 min, followed by an inactivated step at 85 °C for 5 min and an hold step at 4 °C. Quantitative PCR was performed in duplicate for each sample using LightCycler 480^®^ (Roche Applied Science, Germany); a negative control (water) was added for each sample. PCR reactions were carried out in a total volume of 10 μl, consisting of 3 μl of cDNA, 5 μl of Taqman Universal PCR MasterMix (Applied Biosystems) and 2 μl of primer (Life Technologies). The mixture was incubated in a 384-well plate, at 95 °C for 10 min, followed by 50 cycles at 95 °C for 15 s and 60 °C for 1 min. MiR-16, commonly considered as internal control in serum^54^,^55^, was used to normalize the FF miRNA expression levels, due to its constant expression in FF samples (Supplementary Figure S1). The relative expression of the five miRNAs (let-7b, miR-29a, miR-30a, miR-140 and miR-320a) in each FF pool was calculated relative to that of miR-16 by using the equation 2^−∆Ct^, in which ∆Ct was determined by the formula: Ct target miRNA−Ct miR-16.

These five miRNA were chosen among a large panel of miRNAs described in previous studies as expressed in FF^5^,^32^. The potential functions of these miRNAs, their localization in the ovarian follicle and their primary targets are summarized in Supplementary Table S3.

### Statistical analysis

Continuous parametric data are presented as the mean ± standard deviation (SD) and categorical variables with numbers and percentages. Based on the assessment of the normality of the distribution by Shapiro-Wilk test, we used Mann-Whitney tests and Spearman Rank correlations to compare and correlate quantitative variables, respectively. Univariate analyses were performed for each FF miRNA expression and clinical characteristic (reported in Supplementary Table S1), to investigate their associations with PCOS. As FF miR-30a, miR-140 and let-7b expressions were related to PCOS with a p-value lower than 0.05 in univariate analyses, they were integrated in the multivariate analysis. Among the clinical variables, BMI was also correlated significantly with PCOS in the univariate analysis [COR = 1.13 (1.02–1.25), p = 0.02]. Therefore, the adjusted odds ratios for the three miRNAs were calculated by including BMI in the multivariate analysis. The ability of FF miRNA levels to discriminate women with PCOS and to predict blastocyst development and clinical pregnancy outcome was assessed by ROC curves and calculating the AUC with 95% confidence interval (CI). The sensitivity and specificity of the optimal cut-off were calculated. Statistical tests were performed using the R software (version 2.15.2). Results were considered significant when p ≤ 0.05.

## Additional Information

**How to cite this article**: Scalici, E. *et al*. Circulating microRNAs in follicular fluid, powerful tools to explore *in vitro* fertilization process. *Sci. Rep.* **6**, 24976; doi: 10.1038/srep24976 (2016).

## Supplementary Material

Supplementary Information

## Figures and Tables

**Table 1:** Multivariate logistic model showing the association of specific FF mRNAs with polycystic ovary syndrome.

Relative FF microRNAs expression related to PCOS	Univariate analysis		Multivariate analysis	
FF microRNAs	Crude OR [95% CI]	*p-value*	Adjusted OR* [95% CI]	*p-value*
FF miR-30a	4.5 [1.94; 10.57]	p < 0.001	5.0 [1.86; 13.68]	0.001
FF miR-140	0.6 [0.37; 0.96]	0.03	0.52 [0.29; 0.94]	0.03
FF let-7b	1.0 [0.99; 1.0]	0.01	1.0 [0.99; 1.0]	0.02

OR, Odds ratio, FF, follicular fluid, PCOS, polycystic ovary syndrome.

^*^Adjustment for BMI.

**Table 2:** Power of discrimination of FF miRNA expressions for polycystic ovary syndrome prediction.

ROC analysis	Prediction for PCOS			
	FF miR-30a	FF miR-140	FF let-7b	Combination of FF miR-30a, miR-140 and let-7b
AuROC (95% CI)	0.67 (0.57–0.76)	0.67 (0.57–0.76)	0.67 (0.57–0.76)	0.83 (0.73–0.92)
*p-value*	0.02	0.007	0.003	<0.0001
Sensitivity (%)	57.7	57.7	53.9	70.0
Specificity (%)	85.1	81.1	75.7	83.8
Positive predictive value (%)	57.7	51.7	41.9	60
Negative predictive value (%)	85.1	84.5	81.2	88.6
Cut-off value	>0.49	≤0.92	≤93.95	−

MiRNAs were analyzed individually and in combination by Receiver Operating Characteristic (ROC) analysis. AuROC, area under the ROC curve, PCOS, polycystic ovary syndrome.
