# Supplementary material for: Circulating microRNAs in follicular fluid, powerful tools to explore in vitro fertilization process
Source: Sci Rep. 2016 Apr 22;6:24976. doi: 10.1038/srep24976 (PMC4840336; doi:10.1038/srep24976)
Supplement: Supplementary Information [file srep24976-s1.doc]

**TITLE: Circulating microRNAs in follicular fluid, powerful tools to explore *in vitro* fertilization process.**

**Authors: E.Scalici^1,2,3^, S. Traver^1^, T. Mullet^1,2^, N. Molinari^4^, A. Ferrières^3^, C. Brunet^3^, S. Belloc^5^, S. Hamamah^1,2,3*^.**

**Supplementary Table S1:** Clinical characteristics of all patients (n=121) and of each groups: women with normal ovarian reserve (n=91) and with polycystic ovary syndrome (PCOS) (n=30).

SD, standard deviation; BMI, body mass index; FSH, follicle-stimulating hormone; LH, luteinizing hormone; E2, 17β-estradiol; AMH, anti-Müllerian hormone; AFC, antral follicle count; r-FSH, recombinant follicle-stimulating hormone; HP-hMG, highly purified human menopausal gonadotropin *Except four women who received mild stimulation. P-values: Comparisons between women with PCOS and women with normal ovarian reserve; Mann-Whitney test.

**Supplementary Table S2:** IVF outcomes of all patients (n=121) and of each groups: women with normal ovarian reserve (n=91) and with PCOS (n=30).

SD, standard deviation; MII, oocyte blocked in meiotic metaphase II. P-values: Comparisons between women with PCOS and women with normal ovarian reserve; Mann-Whitney test.

**Supplementary Table S3:** Circulating miRNAs expressed in the cumulus-oocyte complex (COC), granulosa cells (GC), cumulus cells (CC) and in follicular fluid (FF): their functions and primary targets in ovarian follicles.

**Supplementary Figure S1:** MiR-16 constant expression between two series of FF samples (n=34) under our experiment conditions.

**
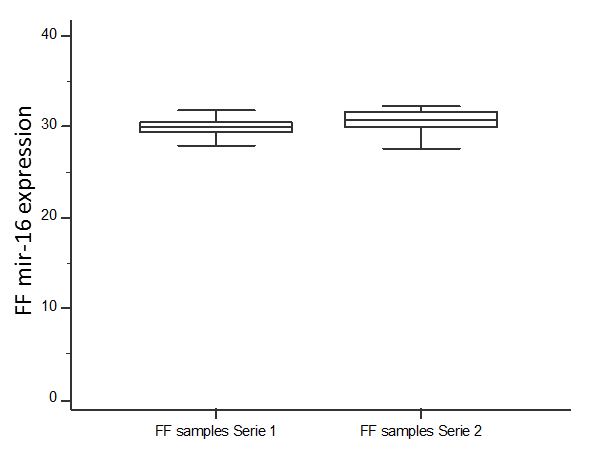
**
